# Multi-locus allelic architecture underlying natural variation in leaf rolling in *japonica* rice

**DOI:** 10.3389/fpls.2026.1839728

**Published:** 2026-08-04

**Authors:** Dongryung Lee, Backki Kim, Joohyun Lee, Soon-Wook Kwon

**Affiliations:** 1 Department of Plant Bioscience, Pusan National University, Miryang, Republic of Korea; 2 Life and Industry Convergence Research Institute, Pusan National University, Miryang, Republic of Korea; 3 Department of Crop Science, Konkuk University, Seoul, Republic of Korea

**Keywords:** cross-validation, japonica rice, leaf rolling, multi-locus allelic combination, multiple linear regression, residual diagnostics

## Abstract

Leaf rolling is a key component of rice canopy architecture that affects light interception, microclimate formation, and planting density. The contribution of naturally occurring allelic variation to quantitative variation in leaf rolling within cultivated rice remains poorly understood, while extreme leaf rolling caused by loss-of-function mutations often results in detrimental pleiotropic effects. Herein, we examined how multi-locus allelic variation contributes to natural variation in leaf rolling within *japonica* rice. Leaf rolling was quantified based on the leaf rolling index (LRI) using a panel of 201 *japonica* accessions. The phenotype was transformed using the Yeo–Johnson method to reduce strong right skewness and improve the distributional properties of the data, thereby facilitating subsequent regression modeling. Haplotype analyses were performed for previously reported leaf rolling-associated genes and genome-wide association study (GWAS) lead loci, leading to the identification of five loci exhibiting substantial haplotype-dependent phenotypic variation. Phenotypically defined allelic groups represented these loci were subsequently evaluated using multiple linear regression (MLR), with the first two principal components derived from genome-wide SNP data included as covariates to account for population structure. The final MLR model identified four loci (*qALR1*, *OsYABBY1*, *OsSLL2*, and *OsSRL10*) as the independent contributors to leaf rolling variation, collectively explaining 21% of the variance in the transformed phenotype after accounting for population structure. Model diagnostics and ten-fold cross-validation supported the statistical validity of the framework and indicated stable model performance across validation folds. Analysis of multi-locus allelic combinations showed 13 distinct configurations that clustered into three phenotypically differentiated groups. This reflected the cumulative dosage of high-leaf rolling alleles. Thus, the natural variation in leaf rolling in *japonica* rice is governed by the additive effects of multiple moderate-impact loci. The multi-locus allelic framework established here provides a statistically sound and biologically interpretable basis for dissecting polygenic canopy traits and practical guidance for developing genetic materials aimed at optimizing rice plant architecture.

## 1 Introduction

The leaf is a main photosynthetic organ that converts solar energy into carbohydrates, thereby facilitating plant growth and productivity. Efficient light interception strongly depends on plant architecture, and leaf rolling is one of the primary traits that shape canopy structure in rice (Ning et al., 2008; Zhi et al., 2022). Breeders have aimed to develop rice varieties with an ideal plant type over the past three decades, in which moderate leaf rolling has been considered beneficial as it stiffens the leaf blade and promotes leaf erectness (Hay et al., 2000; King et al., 1996; Wu, 2009; Yuan, 2017). Such architecture decreases mutual shading, enhances light penetration into lower canopy layers, and ultimately improves whole-plant photosynthetic efficiency (Burgess et al., 2017; Duncan, 1971; Mantilla-Perez and Salas Fernandez, 2017). Additionally, moderate leaf rolling can generate a favorable microclimate along the leaf surface by elevating stomatal resistance and retaining higher humidity, thereby enhancing tolerance to environmental stresses such as drought, high irradiance, and increased temperature (Kadioglu and Terzi, 2007; O’Toole et al., 1979; Sarieva et al., 2010). Furthermore, increased erectness facilitates higher planting density, which can contribute to elevated grain yield (Asmamaw, 2017). Thus, understanding the genetic factors and regulatory mechanisms underlying leaf rolling is necessary for breeding rice varieties with optimized canopy architecture.

Like several agronomic traits, leaf rolling is a complex phenotype shaped by multiple genetic and environmental factors (Cal et al., 2019; Jang et al., 2021). Numerous loci with small to moderate effects influence grain yield, plant height, and tiller number, which are the classic examples of complex traits in rice. Leaf rolling shows similar genetic complexity. To date, > 90 quantitative trait loci linked to leaf rolling have been reported, and genetic studies using mutant and genome-edited lines have identified over 60 genes involved in leaf polarity, bulliform cell formation, sclerenchyma differentiation, commissural vein patterning, and cuticle development (Fu et al., 2019; Lee et al., 2025). However, alleles identified via mutagenesis have rarely been applied in breeding, largely because loss-of function or severe alleles often result in excessive rolling, pleiotropic developmental defects, and decreased plant vigor and yield (Jing et al., 2017; Yuan et al., 2022). Despite extensive functional characterization, whether naturally occurring allelic variation at these genes contributes to the moderate, agronomically desirable forms of leaf rolling noted in cultivated rice remains unclear.

To address this gap, assessing multi-locus allelic combinations provides a promising approach for dissecting complex traits such as leaf rolling. Multi-locus frameworks capture the combined effects of multiple alleles and can identify favorable configurations that facilitate moderate rolling without detrimental pleiotropic effects (Jang et al., 2020; Zhong et al., 2020) unlike single-locus analyses, which explain only a limited proportion of phenotypic variation. In our previous work (Lee et al., 2025), genome-wide association study (GWAS) using a panel composed of *japonica* and *indica* accessions showed a *japonica*-specific association signal. This suggested that the genetic basis of leaf rolling varies between the two subspecies. This result aligns with known morphological and physiological distinctions between *japonica* and *indica*, including the differences in leaf thickness, bulliform cell morphology, and canopy structure, all traits closely associated with leaf rolling (Cal et al., 2019; Chang et al., 2019; Jang et al., 2021; Wang et al., 2023). Based on these subspecies-specific characteristics, the present analysis exclusively focused on the *japonica* accessions to decrease confounding effects and more accurately evaluate how multi-locus allelic variation contributes to leaf rolling.

Herein, we examined how naturally occurring allelic variation across several loci shapes leaf rolling in *japonica* rice. We first defined haplotypes at the key leaf rolling-associated loci and grouped them into functionally interpretable allelic classes. Using these allelic groups, we developed and validated a multiple linear regression (MLR) model that incorporated the first two principal components derived from genome-wide SNP data as covariates to account for population structure, thereby quantifying their individual contributions to phenotypic variation. Subsequently, we assessed the structure and frequency of multi-locus allelic combinations in the *japonica* panel and compared leaf rolling phenotypes across these combinations to evaluate cumulative genetic effects. Thus, these analyses provided an integrated view of the genetic architecture of leaf rolling and supports the identification of allelic configurations that may help in the breeding of rice with enhanced canopy structure.

## 2 Materials and methods

### 2.1 Plant materials and field experiment

We used 201 *japonica* rice (*Oryza sativa* L.) accessions in thisstudy (**Supplementary Table 1**). The *japonica* panel consisted of 177 temperate *japonica*, 22 tropical *japonica*, and two aromatic accessions. All accessions were maintained at the Agricultural Genetic Resource Center, Pusan National University (PNU), South Korea. At the experimental field of PNU, Miryang, South Korea, all plants were grown under irrigated field conditions during the 2022 growing season. Thirty-day-old seedlings were transplanted at a spacing of 15 cm × 30 cm, with one plant per hill, 25 plants per row, and three rows per accession. Throughout the experiment, standard agronomic practices for rice cultivation were applied.

### 2.2 Leaf rolling evaluation and phenotype preprocessing

Leaf rolling was evaluated following the measurement protocol and LRI calculation method described by Lee et al. (2025). Before statistical modeling, the distributional properties of the response variable were assessed using graphical diagnostics and formal statistical tests. Specifically, quantile–quantile (QQ) plots were inspected, and normality was evaluated using the Anderson–Darling (AD) and Jarque–Bera (JB) tests (Anderson and Darling, 1952; Jarque and Bera, 1980). When substantial departures from normality were observed, the response variable (LRI) was transformed using the Yeo–Johnson (YJ) transformation (Yeo and Johnson, 2000) to reduce skewness, improve distributional symmetry, and stabilize variance while accommodating zero values. After transformation, the QQ plots and the AD and JB tests were re-assessed to evaluate changes in distributional properties and confirm improved approximation to normality in the YJ-transformed LRI (YJ-LRI) data prior to subsequent regression analyses.

### 2.3 Haplotype and allelic group analysis

To examine natural variation associated with leaf rolling using LRI data, haplotype and allelicgroup analyses were conducted. Haplotype analysis was performed using sequence variants locatedwithin the promoter, coding, and untranslated regions (UTRs) of 68 previously reported leaf rolling-related genes and loci (**Supplementary Table 2**). All variant calls and genotype information utilized in this analysis were obtained from the dataset described in Lee et al. (2025). Additionally, genomic coordinates of all variants were based on the IRGSP-1.0 reference genome.

For GWAS lead SNPs, accessions were directly grouped according to their allelic state at the leadSNP. For genes with more complex haplotype structures, multi-locus haplotypes were collapsed into two major allelic groups to facilitate phenotypic comparison and regression analysis. In these cases, a representative biallelic tagging SNP that clearly distinguished the two major haplotype clusters was selected, and accessions were assigned to one of the two allelic groups accordingly (**Supplementary Table 1**).

Prior to calculating allelic-group frequencies, accessions with heterozygous calls at the lead or tagging SNP, or with ambiguous haplotype assignments, were excluded during quality control. Allelic groups were retained as independent categories only when their frequency was ≥ 5%. This threshold was selected *a priori* as a pragmatic criterion to restrict group-specific inferences to common allelic groups and to avoid estimating separate effects for sparsely represented categories. In the *japonica* panel, the 5% cutoff corresponded to approximately 10 accessions per group after quality control; groups below this threshold were therefore considered insufficiently represented for stable estimation of group-wise phenotypic means and regression coefficients. Accordingly, low-frequency allelic groups were not interpreted as separate effects, and downstream analyses focused on common allelic groups with adequate representation in the panel.

Phenotypic differences among the haplotypes and/or allelic groups were evaluated using one-way analysis of variance, followed by Tukey’s honestly significant difference test for multiple comparisons where applicable. Loci exhibiting significant group-dependent variation were retained for multiple regression modeling. All analyses were performed using R version 4.5.3 (R Core Team, 2025) using the packages *stats*, *leaps*, *relaimpo*, *caret*, *emmeans*, *multcomp*, *multcompView*, *ggplot2*, and *tidyverse*, as appropriate for regression analyses, cross-validation, model diagnostics, clustering, and visualization.

### 2.4 Multiple linear regression modeling

Genotypic data for the selected leaf rolling-related genes and GWAS lead SNPs were merged with YJ-LRI values. To account for potential population structure within the *japonica* panel, principal component analysis (PCA) was performed using genome-wide SNP markers. The first two principal components (PC1 and PC2) were included as covariates in the downstream regression analysis. Additionally, accessions or loci with > 20% missing data were removed following quality-control procedures commonly adopted in genetic association studies (Anderson et al., 2010; Marees et al., 2018). Multicollinearity among the predictors (i.e., the allelic groups of the selected leaf rolling-related loci) was evaluated using the generalized variance inflation factor (GVIF), with a threshold of 2 (Fox and Monette, 1992). Genotype variables were encoded as categorical factors, and the reference levels were set to the allelic groups with the lowest mean YJ-LRI. A design matrix was generated using the model.matrix function in R and used as the input for the MLR analysis.

Subsequently, the MLR model was fitted using the ordinary least squares (OLS) technique in R (Montgomery et al., 2021), with YJ-LRI as the dependent variable, the allelic groups of five genes as the categorical predictors, and PC1 and PC2 included as covariates to correct for potential subpopulation:


$$Y=\beta_{0}+\sum_{i=1}^{k}\beta_{i}X_{i}+\sum_{j=1}^{m}\gamma_{j}PC_{j}+\epsilon$$

Where *Y* denotes the response variable (the YJ-LRI), *β_0_* is the intercept, *β_i_* represents the estimated effect of each allelic predictor, *X_i_* denotes the categorical predictor variable corresponding to each gene classified into two *i*-th allelic groups, *γ_j_* represents the effect of the *j*-th principal component covariate, *PC_j_* denotes the principal component included to correct for population structure, and *ϵ* is the random error term. Additionally, residual-versus-fitted and QQ plots were examined to assess residual behavior, including the approximate normality and homoscedasticity of the residuals, as part of the model diagnostic procedure. Furthermore, the relative criticality of the independent variables in the linear regression model was quantified using the lmg method implemented in the *relaimpo* package in R (Groemping, 2007).

### 2.5 Model diagnostics and cross-validation of the MLR framework

Residual diagnostics were conducted on the final MLR model as part of the model evaluation process. Normality of the residuals was evaluated using the procedures described above, whereas homoscedasticity was evaluated using the Breusch–Pagan (BP) test (Breusch and Pagan, 1979). Diagnostic plots, including the QQ plots and residuals-versus-fitted plots, were generated for visual inspection. All diagnostic analyses were conducted in R.

A *K*-fold cross-validation (CV) framework was implemented using YJ-LRI as the response variable (Stone, 1974) to assess the predictive performance and generalizability of the MLR model. Samples were randomly partitioned into *K* = 10 folds of approximately equal size. Model performance was quantified using the root mean squared error (RMSE) and the coefficient of determination (*R^2^*) for the transformed and original phenotype scales (Wright, 1921). Performance metrics from the individual folds were averaged to obtain the cross-validated estimates (mean ± standard deviation). All statistical analyses and visualizations were performed in R.

### 2.6 Multi-locus allelic combination analysis

Allelic combinations inferred from the validated genotype data were subjected to clustering analysis to assess their genetic relationships. A pairwise dissimilarity matrix was generated using the Manhattan distance based on the presence or absence of high-leaf rolling alleles at the four loci. Hierarchical clustering was then performed using the unweighted pair group method with arithmetic mean (UPGMA) implemented by the hclust() function from the *stats* package in R (Hibbert, 2017). Cluster boundaries were determined by cutting the dendrogram at empirically defined height (h = 1.5 and h = 1.0), which corresponded to two major groups and three subclusters, respectively, and the three subclusters were utilized for downstream analyses.

### 2.7 Development of allele-specific SNP markers

SNP markers were developed for four leaf rolling-related loci identified via best subsetregression analysis. Allele-specific markers were designed using PRIMER1 for tetra-primeramplification refractory mutation system polymerase chain reaction (You et al., 2015). Genomic DNA extraction and PCR amplification were conducted following the protocols described by (Jang et al., 2021; Murray and Thompson, 1980). **Supplementary Table 3** provides the primer sequences and PCR conditions for each marker.

For the *SRL10* locus, one allele-specific primer set initially failed to producea detectable amplicon. To ensure reliable allele discrimination, an alternative inner forward primerwas redesigned and used for subsequent PCR analysis (**Supplementary Table 3**).

## 3 Results

### 3.1 Differences in leaf rolling among the rice accessions

**Figure 1:**
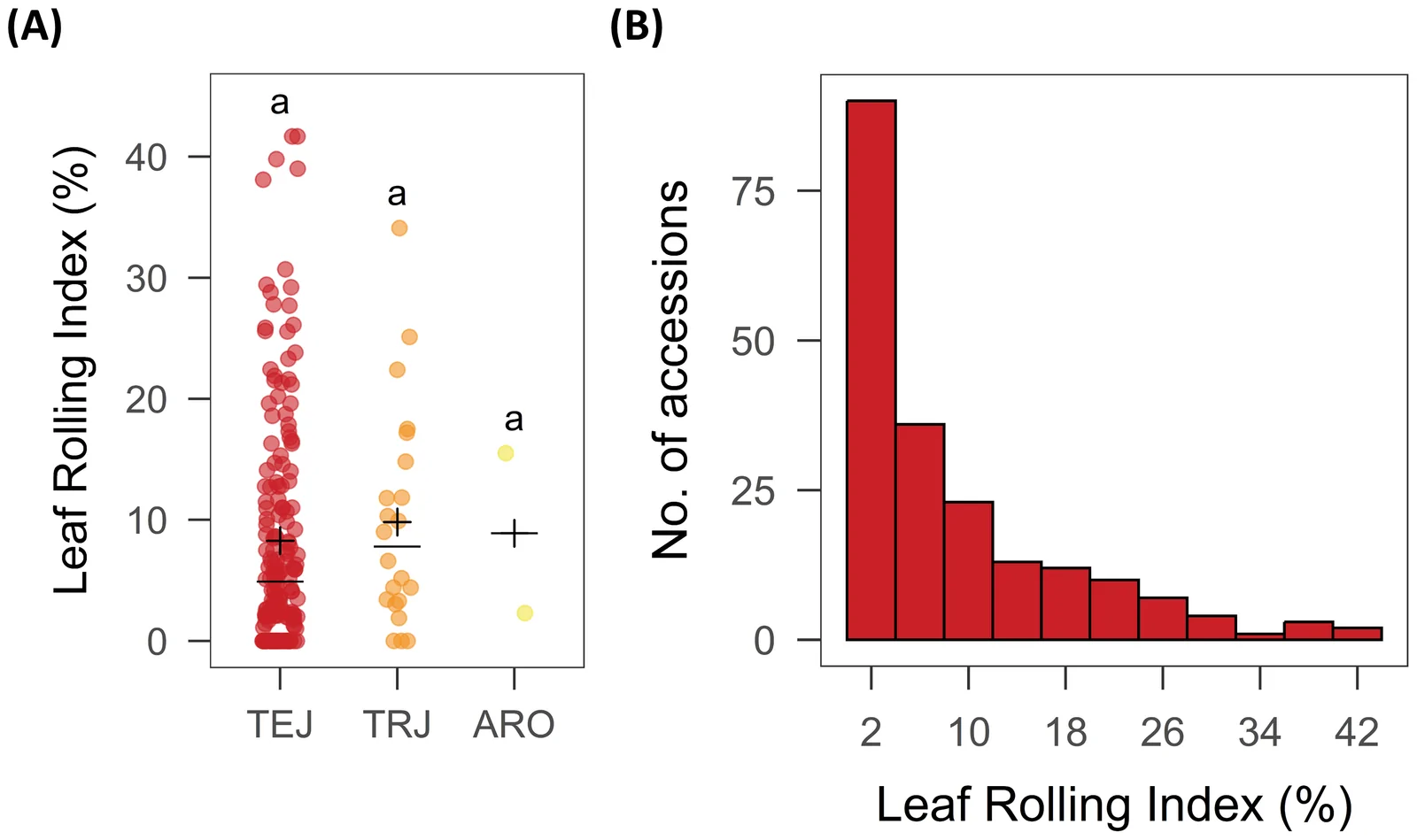
Natural variation and distribution of the leaf rolling index (LRI) among *japonica* rice accessions. **(A)** Variation in the LRI across 201 *japonica* rice accessions, categorized into temperate *japonica* (TEJ), tropical *japonica* (TRJ), and aromatic (ARO) groups. Each point represents an individual accession, and horizontal bars indicate subgroup medians, and crosses denote mean LRI values. Letters indicate the results of multiple comparisons among the subgroups. **(B)** Frequency distribution of the LRI values across the *japonica* panel.

A broad range of variation in leaf rolling was noted among the 201 *japonica* rice accessions, with LRI values ranging from 0% to 41.7%. The overall mean LRI was 8.5%, with average values of 8.3% 9.8%, and 8.9% noted in temperate *japonica*, tropical *japonica*, and aromatic groups, respectively (**Figure 1A**). Mean values slightly differed among the subgroups; however, the differences were statistically insignificant (*p* > 0.05). The LRI distribution was strongly right-skewed, indicating that most *japonica* accessions showed relatively low leaf rolling levels (**Figure 1B**).

### 3.2 Residual diagnostics and phenotype transformation

Before regression analysis, diagnostic tests indicated clear departures from normality in the residual distribution. The QQ plot showed deviations from the theoretical quantiles, primarily in the positive tail (**Supplementary Figure 1A**). The AD and JB tests yielded *p* = 3.7 ×10^-^²^4^ and *p* < 0.001, respectively, supporting this observation. To reduce skewness and improve the distributional properties of the response variable, LRI was transformed using the YJ transformation, which estimated a transformation parameter of λ = 0.075 (**Supplementary Table 1**). Post-transformation diagnostics indicated improved distributional characteristics, with the residual distribution more closely approximating normality; the AD and JB tests yielded *p* = 2.86 × 10^-7^ and *p* = 0.0119, respectively. Although minor deviations from perfect normality persisted, the overall distribution aligned more closely with the theoretical quantiles (**Supplementary Figure 1B**). These results indicate that the transformation effectively reduced skewness and improved residual behavior, thereby facilitating subsequent regression modeling and interpretation.

### 3.3 Identification of leaf rolling-associated loci for MLR analysis

A total of 68 leaf rolling-related genes and GWAS lead SNPs were collected from previous studiesto identify the candidate loci for MLR analysis (**Supplementary Table 2**). Among them, 30 loci were excluded from further analysis because only a single haplotype remained after quality filtering. This was due either to the absence of sequence variation within the present panel or to the exclusion of all alternative haplotypes with frequencies< 5%. Consequently, these loci did not meet the minimum frequency threshold required for reliable haplotype-based comparisons.

**Figure 2:**
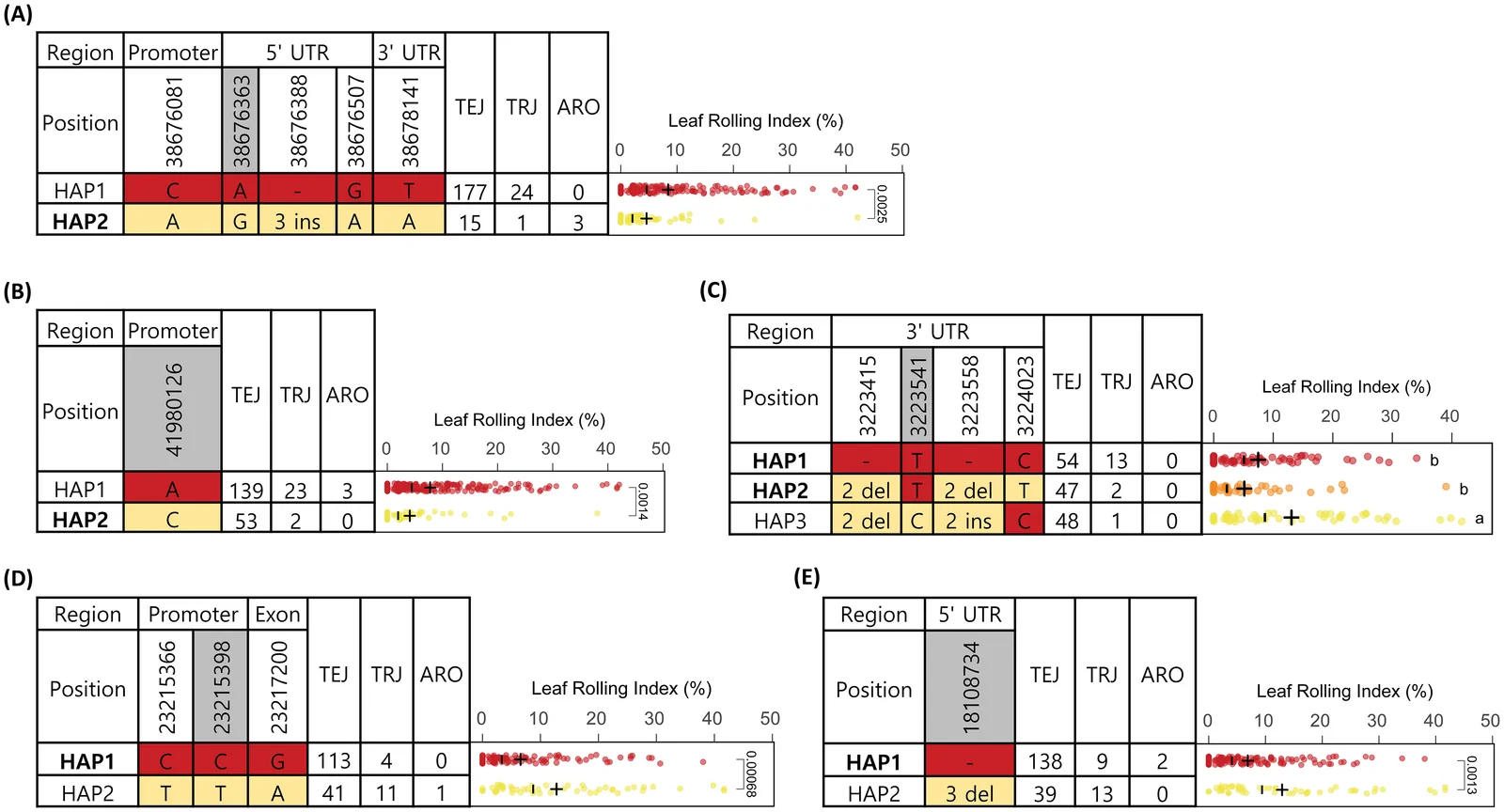
Haplotype definition and phenotypic effects of five leaf rolling-associated loci in *japonica* rice. Haplotype composition, defining genetic variants, and corresponding LRI values are illustrated for **(A)** *OsAS2*, **(B)** *qALR1*, **(C)** *OsYABBY1*, **(D)** *OsSLL2*, and **(E)** *OsSRL10*. For each locus, haplotypes were defined based on the single nucleotide polymorphisms or InDels, with gray-shaded cells indicating representative tagging variants utilized for allelic group classification. Haplotypes indicated in bold denote the low-LRI allelic group and were used as the reference level in subsequent MLR analyses. Scatter plots show the LRI values for individual accessions grouped by haplotype; horizontal bars indicate the median LRI values, and crosses denote mean values. Different letters indicate statistically significant differences among the haplotypes (*p* < 0.05).

Haplotype identification and phenotypic comparison of the remaining 38 loci identified five genes (*OsAS2*, *qALR1*, *OsYABBY1*, *OsSLL2*, and *OsSRL10*) showing statistically significant differences in LRI among haplotypes (**Figure 2**; **Supplementary Table 2**). The genetic variants defining these loci were mainly situated in regulatory regions, including promoter or UTRs, and in one case (*OsSLL2*), a non-synonymous substitution within the first exon was detected. Across loci, accessions were typically grouped into two to three haplotypes, revealing clear separation between high-rolling and low-rolling types. In *OsAS2* and *qALR1*, accessions carrying the major haplotypes (HAP1) showed higher mean LRI values compared with minor haplotypes (**Figures 2A, B**). The high-rolling haplotypes were less frequent but consistently associated with greater leaf rolling for *OsYABBY1*, *OsSLL2*, and *OsSRL10* (**Figures 2C–E**). Overall, these haplotypes revealed consistent associations with LRI variation, indicating that allelic differences at these loci are the major contributors to phenotypic variation in leaf rolling.

**Figure 3:**
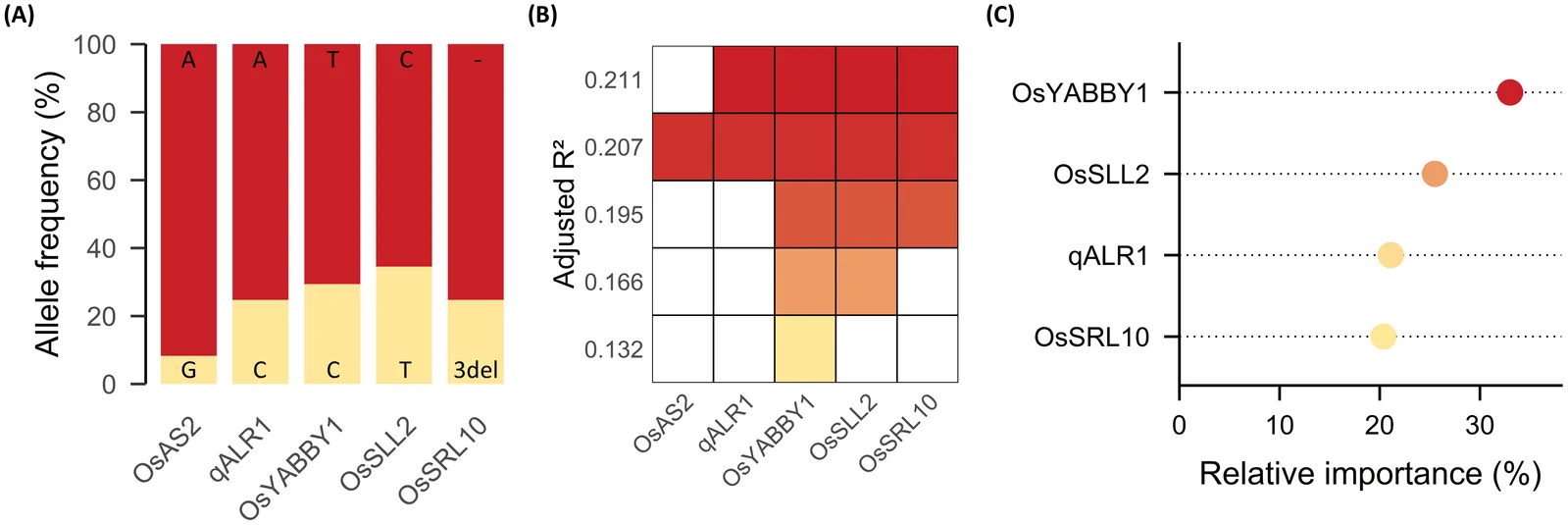
Simplification of haplotypes into representative allelic groups and identification of key predictors contributing to leaf rolling variation. **(A)** Allele frequencies of the representative tagging variants utilized to define two allelic groups for each locus included in the regression analysis. Letters shown within each bar indicate the specific nucleotide or InDel state defining each allelic group. **(B)** Heatmap showing adjusted *R^2^* values for best subset regression models fitted with different predictor combinations. **(C)** Relative importance of the four selected predictors in explaining variation in the YJ-LRI, expressed as the percentage contribution of each predictor to the variance explained by the final regression model.

The haplotypes identified in **Figure 2** were subsequently simplified into two representative allelic groups per locus tagging variants to facilitate downstream MLR analysis and maintain consistency across predictors (**Figure 3A**). In the case of *OsYABBY1*, the three original haplotypes were consolidated into two groups based on their phenotypic similarity (**Supplementary Figure 2**). Haplotypes carrying the T allele at position 3,223,541 consistently showed lower LRI values than those in haplotypes carrying the C allele (**Figure 2C**); therefore, these haplotypes were combined into a single allelic group for the MLR analysis.

### 3.4 MLR-based detection of the loci contributing to natural variation in leaf rolling

Five loci showing significant haplotype-dependent differences in YJ-LRI were selected as candidate predictors for subsequent regression analyses. Before model fitting, multicollinearity among these predictors was evaluated to assess whether their effects could be estimated independently. All predictors showed extremely low GVIF values (1.051-1.266), indicating negligible multicollinearity. Consistently, a correlation heatmap of dummy-coded genotype variables revealed only weak pairwise corrections among the loci (**Supplementary Figure 3**), supporting their inclusion in the same regression model.

To account for potential population structure within the *japonica* panel, genome-wide PCA was performed and the first two principal components were included as covariates. PC1 and PC2 explained 18.69% and 8.78% of the total genetic variance, respectively (**Supplementary Figure 4**). Based on locus-specific differences in mean YJ-LRI, Group2 was used as reference level for *OsAS2* and *qALR1*, whereas Group1 was used as the reference level for *OsYABBY1*, *OsSLL2*, and *OsSRL10* (**Figure 2**; **Supplementary Figure 2**).

An MLR model incorporating PC1 and PC2 as covariates explained approximately 21% of variation in the transformed phenotype (**Table 1**). Four loci, *qALR1*, *OsYABBY1*, *OsSLL2*, and *OsSRL10*, exhibited significant positive effects on leaf rolling, whereas *OsAS2* showed a small and non-significant effect. These results suggest that allelic variation at *qALR1*, *OsYABBY1*, *OsSLL2*, and *OsSRL10* is associated with increased leaf rolling within the *japonica* panel after accounting for population structure covariates.

**Table 1:** Multiple linear regression model for YJ-LRI in rice.

Loci	Binary code of allele		Estimate	Standard error	T-value
	0	1			
(Intercept)			0.97	0.31	3.15**
OsAS2	G (L)	A (H)	0.05	0.30	0.15^ns^
qALR1	C (L)	A (H)	0.44	0.21	2.16*
OsYABBY1	T (L)	C (H)	0.76	0.19	4.04***
OsSLL2	C (L)	T (H)	0.49	0.19	2.59*
OsSRL10	- (L)	3 del (H)	0.54	0.21	2.59*
PC1			0.0003	0.0004	0.76
PC2			-0.0007	0.0006	-1.21
Regression equation: YJ−LRI=0.97+0.05 (OsAS2)+0.44 (qALR1)+0.76 (OsYABBY1)+0.49 (OsSLL2)+0.54 (OsSRL10)+0.0003 (PC1)–0.0007 (PC2)					
*R^2^*	0.2355				
Adjusted *R^2^*	0.2068				
F-statistic	8.186				
p-value	< 0.001				

L and H denote allelic groups associated with low and high YJ-LRI, respectively. Regression coefficients represent estimated effects relative to the low YJ-LRI allele (L). *ns*, not significant; *, *p* < 0.05; **, *p* < 0.01; ***, *p* < 0.001.

Best subset regression, performed while retaining PC1 and PC2 as fixed covariates, identified the same four loci (*qALR1*, *OsYABBY1*, *OsSLL2*, and *OsSRL10*) as the most informative predictors of YJ-LRI variation (**Figure 3B**). Notably, exclusion of *OsAS2* resulted in a slightly higher adjusted *R^2^* (0.211) than that of the full five-locus model, indicating that a more parsimonious four-locus model was sufficient to capture the genetic variation explained by the regression framework. Relative importance analysis based on the population structure-corrected model revealed that *OsYABBY1* contributed the largest proportion of the explained variance (33.0%), followed by *OsSLL2* (25.5%), *qALR1* (21.1%), and *OsSRL10* (20.4%) (**Figure 3C**). Thus, after accounting for population structure, these four loci captured the genetic component of variation explained by the regression model.

### 3.5 Model diagnostics and cross-validated predictive performance

Residual diagnostics were conducted for the best-subset MLR model including the four allelic-group predictors as part of the model diagnostic procedure (**Supplementary Figures 5A, B**). Normality tests indicated minor departures from normality, with the AD test yielding *p* = 0.00908 and the JB test yielding *p* = 0.0624. No evidence of heteroscedasticity was noted by the BP test (*p* = 0.846). Collectively, these diagnostics indicate acceptable residual behavior, with no evidence of heteroscedasticity and only minor departures from normality, providing support for the interpretation of the subsequent regression analyses.

Ten-fold CV was conducted to evaluate model stability and assess potential overfitting. The model revealed consistent predictive performance across folds, with tightly clustered RMSE values (mean = 1.16) and positive *R^2^* values averaging approximately 0.105 (**Supplementary Figures 5C, D**). The low variability of these metrics across folds supports the stability of the fitted model and suggests no clear evidence of overfitting.

### 3.6 Allelic combinations of leaf rolling-related genes

We genotyped all accessions using the developed markers, including the optimized marker for the *OsSRL10* locus, and confirmed that the marker calls were fully consistent with the corresponding variants identified from the resequencing data (**Supplementary Figure 6**). This complete concordance demonstrates that the developed markers reliably discriminate the target alleles in the present panel and provides a practical basis for subsequent genetic analyses and potential marker-assisted breeding applications.

**Figure 4:**
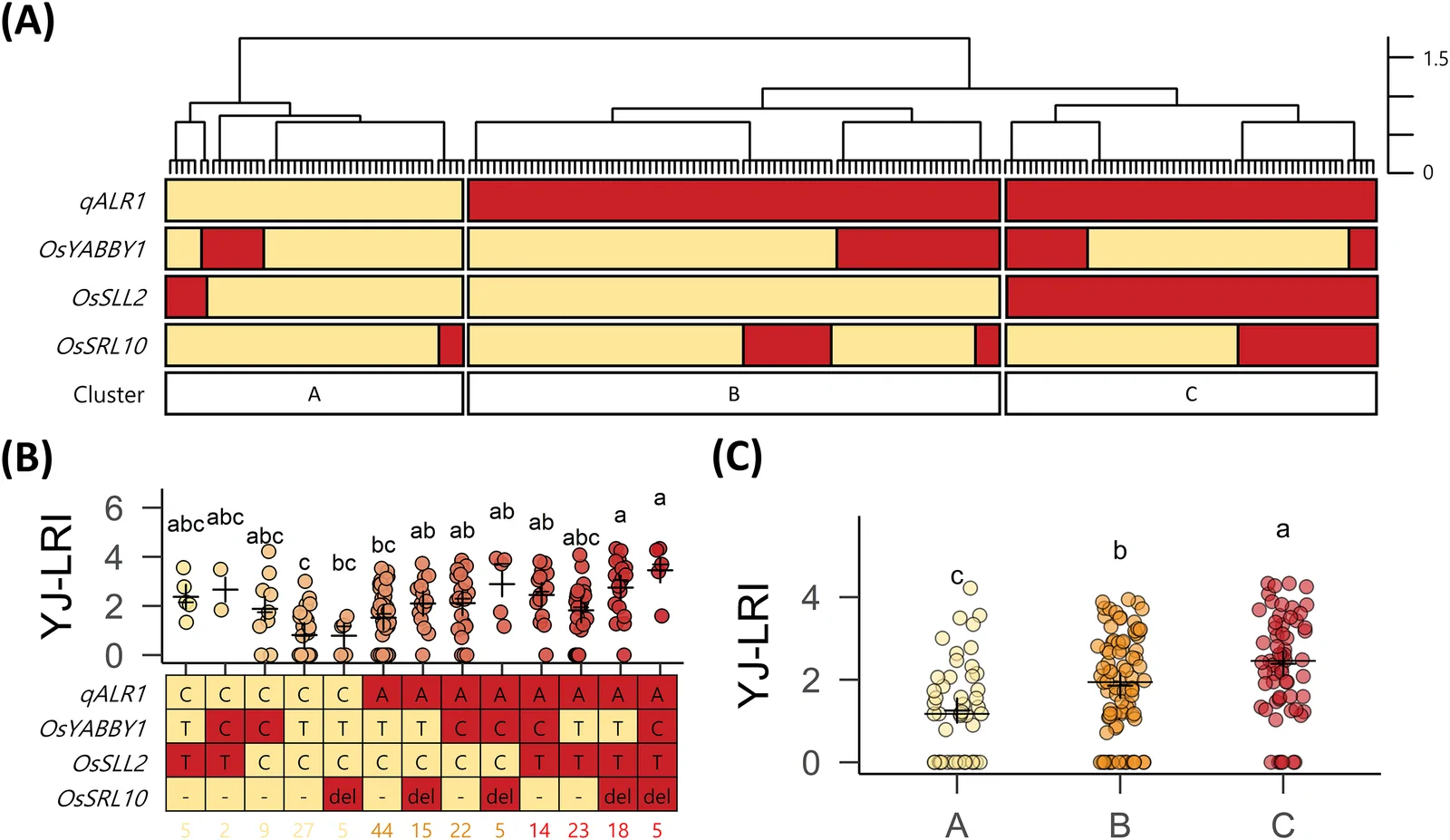
Multi-locus allelic combinations across four leaf rolling-associated loci and their phenotypic effects on the YJ-LRI. **(A)** UPGMA dendrogram constructed from pairwise Euclidean distances based on the presence or absence of high-leaf rolling alleles at the four loci. Red bars indicate alleles associated with high YJ-LRI, whereas yellow bars indicate alleles associated with low YJ-LRI **(B)** Composition and frequency of the 13 allelic combinations, revealing the specific allelic states at each locus and the number of accessions per combination. The colors of the accession counts indicate the corresponding allelic clusters, with yellow, orange, and red representing Cluster A, Cluster B, and Cluster C, respectively. **(C)** Distribution of the YJ-LRI values for accessions grouped by allelic cluster **(A–C)**. Points represent individual accessions; horizontal bars indicate median values, and crosses denote means. Different letters indicate statistically significant differences among the clusters (*p* < 0.05).

Using these validated genotypes, we identified 13 allelic combinations across the panel (**Figures 4A, B**). UPGMA clustering of these combinations showed three subclusters at a height near 1.0, the latter of which were designated as Clusters A, B, and C for downstream analyses. Cluster A was characterized by the uniform presence of the low-leaf rolling C allele at *qALR1*, whereas Clusters B and C predominantly carried the high-leaf rolling A allele at this locus. Similarly, all accessions assigned to Cluster C possessed the T allele of *OsSLL2*, which is associated with increased leaf rolling. Mean YJ-LRI values progressively increased from Cluster A to Cluster C, and significant differences were noted among the three clusters (**Figure 4C**). Consistently, accessions in Cluster A, B, and C carried, on average, one, two, and three high-leaf rolling alleles, respectively (**Figure 4B**). Notably, the fourth and thirteenth allelic combinations exclusively consisted of either low-rolling or high-rolling allelic groups and showed markedly different YJ-LRI values.

Thus, these findings show that combinations of alleles at the four loci clearly stratify *japonica* accessions into phenotypically distinct clusters, reflecting the cumulative effects of high-leaf rolling alleles and illustrating the multi-locus genetic architecture underlying natural variation in leaf rolling.

## 4 Discussion

### 4.1 Impact of phenotype transformation on model assumptions and predictive reliability

Residual normality is commonly evaluated in regression analyses because severe departures from normality can affect statistical inference and the interpretation of estimated effects (Montgomery et al., 2021). However, OLS regression is generally robust to moderate deviations from normality, particularly in datasets of the size used in this study. Therefore, the objective of phenotype transformation was not to establish the validity of the regression framework itself, but rather to improve model behavior and residual distribution and overall model behavior.

The initial LRI values showed substantial right skewness (**Figure 1B**), resulting in residual distributions that deviated from normality when analyzed on the raw scale (**Supplementary Figure 1A**). To reduce skewness and stabilize the residual distribution while accommodating zero values, the YJ transformation was applied (Yeo and Johnson, 2000). The transformed phenotype produced residuals that more closely approximated a normal distribution than the raw phenotype, as supported by graphical diagnostics (**Supplementary Figure 1B**) and formal statistical tests.

Although minor departures from normality remained, the final regression model fitted using the YJ-transformed phenotype exhibited acceptable residual behavior (**Supplementary Figures 5A, B**). Together with the absence of heteroscedasticity, these diagnostics support the use of the model for subsequent analyses. While application of the model to new accessions requires consistent transformation of phenotypic values, this represents a minor practical consideration compared with the benefits of improved residual characteristics.

### 4.2 Evidence-based selection of the genetic predictors for MLR modeling

Selecting a well-defined set of predictors is crucial for constructing interpretable MLR models, because including an excessive number of loci can inflate model complexity, decrease statistical power, and obscure the effects of biologically meaningful variants via redundancy or noise (Montesinos López et al., 2022). We mitigated these risks by applying a multi-step filtering framework to refine the predictor set before model fitting.

Among the previously reported leaf rolling-associated loci (**Supplementary Table 2**), only a small proportion showed meaningful haplotype variation and substantial phenotypic differentiation within the *japonica* panel (**Figure 2**). Consequently, subsequent analyses focused on loci that contributed informative genetic variation within the population under study. Although this approach may have excluded some rare alleles with potentially important biological effects, it enabled subsequent analyses to focus on loci that were sufficiently represented for more stable estimation of their effects within the present panel. Additional studies using larger and more diverse germplasm collections may help clarify the contribution of rare alleles to natural variation in leaf rolling.

Each locus was represented by two allelic groups defined through representative tagging variants to harmonize predictor structure and prevent unnecessary dimensionality (**Figure 3A**). This strategy preserved the necessary genetic signal while maintaining a compact and interpretable predictor framework suitable for regression modeling. Multicollinearity assessment confirmed that the resulting five predictors largely contributed to independent information (**Supplementary Figure 3**), fulfilling the essential requirement for stable regression coefficient estimation.

Collectively, this predictor selection process established an analytical foundation for subsequent MLR analyses, facilitating clear assessment of locus-specific contributions while maintaining statistical stability and biological interpretability. This framework provides a rational starting point for evaluating how genetic predictors affect model adequacy and explain natural variation in leaf rolling by systematically constraining model complexity and focusing on informative genetic variation.

### 4.3 Genetic predictors enhance model adequacy and explain natural variation in leaf rolling

The final regression model explained approximately 21% of the variation in the transformed phenotype (**Figure 3B**), although a substantial fraction remained unexplained. This outcome is not unexpected for a complex quantitative trait (Ali et al., 2016; Jang et al., 2020; Mackay, 2001; Matsubara et al., 2016), which is likely influenced by numerous additional loci of small effect, gene-gene interactions, and environmental factors. Therefore, the loci identified in this study should be regarded as important contributors to natural variation in leaf rolling rather than a complete representation of its underlying genetic architecture.

The relative contributions of multiple loci (**Figure 3C**), rather than the dominance of a single locus, support a polygenic basis for leaf rolling variation. This finding highlights the importance of considering the combined effects of multiple genes when investigating the genetic architecture of this trait.

The present framework should be interpreted as a biologically informed multi-locus regressionanalysis rather than a genome-wide mixed-model association approach. Only *qALR1* wasderived from GWAS signals, whereas the remaining predictors were selected from previously characterized leaf rolling-related genes that exhibited significant haplotype-dependent phenotypic variation in the *japonica* panel (**Supplementary Table 2**). Accordingly, OLS-based MLR was used to estimate the independent and combined effects of a biologically supported set of loci while maintaining model interpretability.

Although PC1 and PC2 were included as covariates to account for population structure, kinship was not explicitly modeled as a random effect. This represents a limitation of the current framework, and future studies using larger populations and mixed-model approaches may further refine estimates of locus-specific effects. Nevertheless, the four-locus framework provides a useful basis for investigating how naturally occurring allelic variation contributes to leaf rolling and may facilitate future breeding efforts aimed at optimizing rice plant architecture.

### 4.4 Allelic combinations and their breeding implications

The analysis of multi-locus allele combination showed how multiple leaf rolling-associated loci collectively contribute to phenotypic variation. Naturally occurring allele combinations showed distinct frequencies and clear phenotypic differences (**Figure 4B**), and UPGMA clustering provided a structured summary of these patterns (**Figure 4A**). The three resulting clusters reflected an additive elevation in the number of high-leaf rolling alleles and revealed clear separation in the YJ-LRI values (**Figure 4C**). Thus, phenotypic structure not readily apparent from single-locus analyses can emerge when loci are considered in combination. This finding highlights the criticality of accounting for the cumulative effects of multiple moderate-impact loci when interpreting the genetic architecture of polygenic traits such as leaf rolling.

Leaf rolling has been proposed to affect canopy architecture, light interception, transpiration, and potentially grain yield (Baret et al., 2018; Sinclair and Sheehy, 1999). However, direct evidence linking moderate levels of leaf rolling to enhanced physiological performance or agronomic productivity remains scarce. Rigorous assessment of such effects needs genetic materials that specifically vary at leaf rolling-associated loci, allowing phenotypic consequences to be evaluated independently of the background genetic variation. To date, much of the existing knowledge has been derived from ideal plant architecture models or studies performed under specific stress conditions (Cal et al., 2019; Khush, 2013; Wu, 2009), which may not completely reflect the outcomes under typical field environments. Consequently, the agronomic significance of naturally occurring variation in leaf rolling within *japonica* germplasm remains insufficiently resolved.

In this context, the multi-locus allelic clusters identified in this study provide a valuable starting point for future physiological and agronomic examinations. Given that these clusters vary in the cumulative dosage of high-leaf rolling alleles, they provide a practical framework for selecting contrasting genetic backgrounds that specifically differ at the leaf rolling loci. The allele combinations defined here help identify the most informative sets of alleles for generating such materials, although further genetic refinement through backcrossing or the development of near-isogenic lines will be required to minimize background effects. Moreover, the allele-specific markers developed in this study showed complete concordance with the corresponding resequencing variants in the validation panel, supporting their reliability for allele discrimination. Although broader validation across additional germplasm collections and breeding populations will be valuable, these markers provide practical tools for identifying favorable alleles and facilitating marker-assisted selection. More broadly, the allelic frameworks established in this study provides a foundation for testing whether natural variation in leaf rolling provides measurable agronomic advantages and for guiding breeding strategies aimed at optimizing canopy architecture in rice.

## 5 Conclusion

This study provides evidence that natural variation in leaf rolling within *japonica* rice is governed by the cumulative and additive effects of multiple moderate-impact loci rather than by a single major gene. We established a statistical framework that identified several major genetic contributions to leaf rolling by integrating phenotype transformation, evidence-based predictor selection, and multi-locus analyses. The multi-locus allelic combinations identified here clarify how multiple loci jointly shape phenotypic variation and provide informative genetic contrasts that can guide targeted material development for future physiological and agronomic assessment. Collectively, these insights provide a foundational resource for determining the functional importance of leaf rolling and for informing subsequent research aimed at understanding its potential agronomic implications.

## Data Availability

The data supporting the findings of this study are included in the article and its **Supplementary Material**. Further inquiries can be directed to the corresponding authors.

## Supplementary material

Supplementary Figure 1 QQ plots of standardized residuals before and after YJ transformation. **(A)** QQ plot of standardized residuals based on the raw LRI values. **(B)** QQ plot of standardized residuals after YJ transformation (λ = 0.075). Shaded areas indicate the 95% confidence envelope.

Supplementary Figure 2 Phenotypic basis for consolidating *OsYABBY1* haplotypes into two allelic groups.

Supplementary Figure 3 Correlation structure among allelic group-derived genetic predictors for leaf rolling. Heatmap showing pairwise Pearson correlation coefficients among dummy-coded genotype variables corresponding to the five selected loci. Color intensity reflects the magnitude and direction of correlation, with values indicated within each cell.

Supplementary Figure 4 Population structure of the *japonica* panel based on genome-wide principal component analysis. Principal component scores are plotted using PC1 and PC2. Points represent individual accessions and are colored according to subgroups classification: temperate *japonica* (TEJ), tropical *japonica* (TRJ), and aromatic (ARO).

Supplementary Figure 5 Residual diagnostics and predictive performance of the best-subset MLR model including four allelic-group predictors. **(A)** Normal QQ plot of standardized residuals from the final MLR model fitted to the YJ-LRI. **(B)** Plot of standardized residuals versus fitted values. **(C)** Distribution of RMSE values obtained from ten-fold CV. **(D)** Distribution of CV *R^2^* values across folds.

Supplementary Figure 6 Validation of allele-specific markers for allelic group discrimination. Allele-specific PCR markers were used to distinguish allelic groups at four loci: **(A)** *qALR1*, **(B)** *OsYABBY1*, **(C)** *OsSLL2*, and **(D)** *OsSRL10*. In **(D)**, lanes 1–2 and 3–4 represent non-deletion and 3-bp deletion alleles, respectively, using the original primer set, whereas lanes 5–6 show successful amplification of the deletion allele using an additional outer forward primer. Labels below each gel indicate the corresponding allelic groups.
